# The whole is greater than its parts: ensembling improves protein contact prediction

**DOI:** 10.1038/s41598-021-87524-0

**Published:** 2021-04-13

**Authors:** Wendy M. Billings, Connor J. Morris, Dennis Della Corte

**Affiliations:** Department of Physics and Astronomy, Brigham Young University, Provo, UT USA

**Keywords:** Biological physics, Computational methods

## Abstract

The prediction of amino acid contacts from protein sequence is an important problem, as protein contacts are a vital step towards the prediction of folded protein structures. We propose that a powerful concept from deep learning, called ensembling, can increase the accuracy of protein contact predictions by combining the outputs of different neural network models. We show that ensembling the predictions made by different groups at the recent Critical Assessment of Protein Structure Prediction (CASP13) outperforms all individual groups. Further, we show that contacts derived from the distance predictions of three additional deep neural networks—AlphaFold, trRosetta, and ProSPr—can be substantially improved by ensembling all three networks. We also show that ensembling these recent deep neural networks with the best CASP13 group creates a superior contact prediction tool. Finally, we demonstrate that two ensembled networks can successfully differentiate between the folds of two highly homologous sequences. In order to build further on these findings, we propose the creation of a better protein contact benchmark set and additional open-source contact prediction methods.

## Introduction

The function of proteins is directly related to their structure. It is much easier to identify protein sequences than structures, as reflected by the number of available sequences (~ 195 M^1^) and deposited structures in the protein data bank (~ 170 k^2^). The prediction of protein structure from primary sequence is a long-standing challenge that has recently seen major advancements through two-stage folding pipelines^3^. Such two-stage methods first predict one- and two-dimensional protein structure annotations^4^ (PSAs), such as amino acid contact or distance probabilities, using machine learning methods. Then, they construct an atomistic model under the constraints of such PSAs.

One important PSA which is considered sufficient to construct a protein structure^5^ is the set of contacts between pairs of amino acids in a folded protein. The biannual Critical Assessment of Protein Structure Prediction (CASP) defines two nonadjacent amino acids to be in contact if the Cβ (or Cα in the case of glycine) distance is less than 8 Å in the folded structure. CASP has assessed the progress in method development for contact predictions since its 2nd installment in 1996^6^. In CASP13 (held in 2018), 46 groups participated in the contact prediction challenge with varying degrees of success^7^.

While individual groups have focused mainly on developing superior stand-alone solutions, we introduce the powerful concept of ensembling^8^–often used in deep learning—to this challenge. A large corpus of literature shows that ensembling deep learning methods improves predictions, as it limits the common trap of overfitting by training diverse networks on the same small dataset^9–11^. A recent review by Cao et al*.* illustrates various examples of the ensembling technique applied within bioinformatics^12^. Successful CASP13 groups, including RaptorX^13^, TripletRes^14^, and AlphaFold^15^ used ensembling within their own models, but no one has yet investigated the effects of ensembling entirely different contact prediction methods. We hypothesize that a combination of protein contact prediction networks will produce better results than any individual model. Therefore, rather than treating individual groups as stand-alone competitors, we suggest ensembling the predictions of various groups to explore whether an ensemble could outperform individual entrants.

Since CASP13, several new tools have emerged that focus on predicting amino acid distances rather than contacts. It is possible to convert distances into contact predictions (see Methods), so we also compared the performance of three new deep learning methods (AlphaFold^15^, trRosetta^16^, and ProSPr^17^) that did not contribute to the CASP13 contact assessment with participating groups, both individually and as an ensemble.

This paper first evaluates the impact of ensembling on the groups that contributed to CASP13 contact prediction. Second, it converts distance predictions of the three deep networks into contacts and shows that these methods are superior contact predictors. Third, it shows that even the best standalone network benefits from ensembling with additional, slightly inferior predictions from unrelated methods. We call for a better standardized training and test set to select ideal ensemble methods, and for additional groups to contribute easily reusable network models to enable ensembling over a larger variety of methods.

## Materials and methods

### Obtaining predictions from various groups

Contact predictions made by groups that participated in the CASP13 contact evaluation were downloaded from the CASP archive^18^. AlphaFold distance predictions for CASP13 targets were released after the competition and obtained online^19^. We created ProSPr predictions for the same targets (see Data Availability) in accordance with the CASP13 sequences using our most recently developed models. We obtained trRosetta distance predictions for the same sequences using the online server (July 2020 version)^16^. Rather than using the post-prediction domains defined by CASP for evaluation (not available to predictors during the competition), we chose to conduct our analysis using predictions made for the entire sequence of each target. This decision was informed in part by the strict availability of only target-level predictions for AlphaFold, as well as inconsistencies in the reported domain mappings.

### Building the label set

We constructed our label set based on the PDB structures of CASP13 targets that have been made publicly available at the time of this study^20^. Each structure was downloaded from the RCSB PDB^2^ and adjusted to maximize agreement with the CASP13 target sequence the predictors received. For example, residues in the structure that disagreed with the prediction sequence were removed for analysis. We then calculated the distances between the Cβ atoms (or Cα in the case of glycine) for each pair of amino acids in the structure. Residues separated by 8 Å or less were defined as contacts for the given target. Edited PDB structures, distance matrices, and contact label lists are made available with this work (see Data Availability).

### Definition of contact classes

Throughout this work contacts of different ranges refer to the sequence separation of two residues that are found or predicted to be in contact. Amino acid contacts were grouped according to their sequence separation into short, mid, and long categories, defined as pairs of residues separated by 6 to 11, 12 to 23, and 24 + residues, respectively^7^.

### Deriving contacts from distance predictions

**Figure 1:**
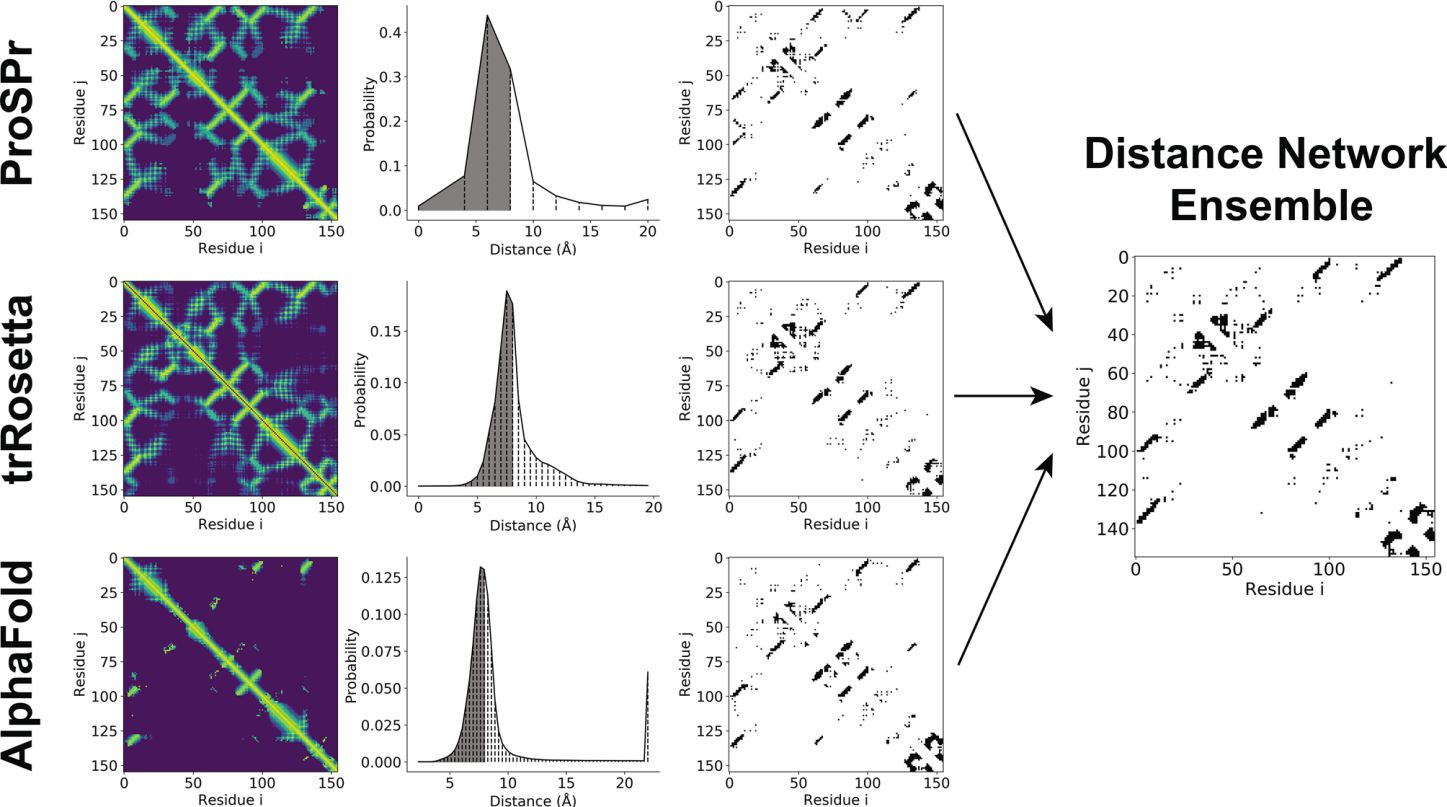
Schematic of method for deriving contacts from pairwise amino acid residue distance predictions and creating an ensemble, showing CASP13 domain T0986s2 as an example. The first column shows a flattened representation of the predictions from each of the three networks (ProSPr, trRosetta, and AlphaFold) where the brightness of each pixel represents how close the pair is most likely to be. The middle column shows an example of the full distance distribution for a specific amino acid pair (i = 130, j = 9) with vertical dashed lines showing the bin divisions used by each model (10, 36, and 64 bins respectively), and the shading representing the total probability to be in contact (distance ≤ 8 Å). Finally, the third column shows the most likely *L* contacts for each of the three ranges predicted by each network and provides comparison to the set of contacts predicted by ensembling the three networks.

Distance predictions were converted into contacts using the procedure outlined in Fig. 1. The predictions from each of the three models (ProSPr, trRosetta, AlphaFold) reported a probability distribution over distance for each amino acid pair, as depicted in the second column. One major difference between the three networks is the number of bins dividing the distance range, and the span of the range. To obtain a contact probability for each residue pair from these distributions, the probabilities of all distance bins up to 8 Å were summed. For ProSPr and trRosetta this corresponds to bins 0–2 and 1–12, respectively. For AlphaFold the probabilities of bins 0–18 and 20% of bin 19 were summed, as AlphaFold does not have a bin division exactly at 8 Å; this also reproduces the contact probabilities reported by AlphaFold^15^.

The contact probabilities for all amino acid pairs were divided into short, mid, and long contact classes. The most probable *L* (sequence length) contacts in each class were considered to be in contact. The result of this step is depicted in the third column of Fig. 1.

### Constructing ensembles

To create ensembles of predictions, we averaged the contact probabilities for each residue pair from the probabilities predicted by the individual methods and selected the most likely *L* contact pairs in each of the short, mid, and long ranges. An example of this result is shown for CASP13 target T086s2 in Fig. 1.

### Calculating accuracy scores

We calculated the average accuracy of each method on the 41 CASP13 targets that composed the intersection of the predictions obtained (limited availability of AlphaFold distance predictions) and the label set of contacts derived from structures in the PDB (limited number of published CASP13 targets). The equation for calculating contact accuracy reduces to the definition of precision, as we do not make negative predictions. This definition has been used previously in other works^21^, and is given as:

1 $$\mathrm{Accuracy}= \frac{\mathrm{TP}+\mathrm{TN}}{\mathrm{TP}+\mathrm{FP}+\mathrm{FN}+\mathrm{TN}}=\mathrm{ Precision }= \frac{\mathrm{TP}}{\mathrm{TP}+\mathrm{FP}}$$

The accuracy for the three contact categories was first calculated for each target. Not all proteins have at least *L* short, mid, and long contacts. Therefore, the possible accuracy score does not range from 0 to 1, but rather has a lower ceiling. We normalized all average accuracy scores with regard to the respective ceiling for each range to show results on a range from 0 to 1. The average accuracies for a method are the average over all contact categories.

### Jaccard distance

The Jaccard distance ranges from 0 (identical sets) to 1 (no similarity) and was used to analyze the similarity of predicted contacts between groups and ensembles. The Jaccard distance (*d*_*j*_) between two sets of contacts, A and B, is calculated as:

2 $$d_{j} = \frac{{\left| {A \cup B} \right| - \left| {A \cap B} \right|}}{{\left| {A \cup B} \right|}}$$

### Applying the ensemble to a test case of two similar sequences

Contact labels were derived from the average distance matrices of NMR models contained in PDB files 2KDL and 2KDM. The same HHBlits multiple sequence alignments, generated by the trRosetta server, were used to create distance predictions with both the ProSPr and trRosetta networks (see Data Availability). Prediction ensembling was then conducted as described in “Constructing ensembles” section and scored according to the method outlined in “Calculating accuracy scores” section. While we cannot verify whether the two sequences were used during training of trRosetta, they were not included in the training of ProSPr.

## Results and discussion

### Proof of principle–ensembling CASP13 contact predictions

**Figure 2:**
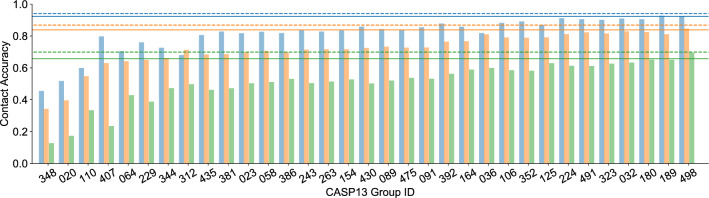
Comparison of average contact prediction accuracies for groups from the CASP13 RR category. Accuracies for short, mid, and long contact ranges are shown in blue, orange, and green, respectively. Group IDs can be mapped to authors using the CASP13 groups page^25^. Solid lines represent the average accuracies of predictions made with an ensemble of all 33 groups shown. Dashed lines show average prediction accuracies for an ensemble consisting of only the top 20% of all groups shown (7 right groups) as determined by average contact accuracy across all three ranges.

We initially tested the concept of ensembling using all groups that submitted contact predictions to the CASP13 competition for our entire test set of 41 targets. Figure 2 shows the respective prediction accuracies for each qualifying group on short-, mid-, and long-range contacts (defined in Methods). To illustrate the effects of ensembling, we also present the respective scores of two ensembles built from the submissions. The solid lines indicate that an ensemble of all groups improves accuracy for all except the winning group 498 (RaptorX). The dashed lines show that ensembling only the top 20% of CASP13 groups improves the accuracy for even the best group, in all three contact ranges.

Based on this analysis, we propose that superior contact predictions are achievable not only through improved methods, but also by incorporating democratized predictions from other methods. If it were possible to easily predict contacts with various tools, an ensemble would likely always be better than the output of a single prediction tool.

### Going deeper–ensembling deep learning distance prediction networks

To test our hypothesis further, we decided to compare the contact accuracies of recent deep neural networks that were released after CASP13 and predict pairwise amino acid distances. For three important networks with available distance predictions for CASP13 targets, we converted the distances into contacts and assessed performance. The prediction accuracies for each of these networks (shown in Table 1) are in all cases superior to CASP13 winner RaptorX in at least one category (short, mid, or long). AlphaFold contact predictions on this test set are superior to all other individual networks in all three categories.

**Table 1:** Contact accuracies for different prediction methods evaluated on CASP13 test set (largest column value in bold).

Method	Short (%)	Mid (%)	Long (%)	Average (%)
RaptorX (498)	92.47	84.65	69.46	82.19
ProSPr	93.12	83.10	67.21	81.14
trRosetta	92.73	84.77	69.30	82.27
AlphaFold	95.13	87.12	72.53	84.93
Distance Network Ensemble^†^	**95.97**	87.58	74.16	85.91
Overall Top 4 Ensemble^‡^	95.58	**87.98**	**74.67**	**86.08**

^†^Combines ProSPr, trRosetta, and AlphaFold predictions.

^‡^Combines predictions from ProSPr, trRosetta, AlphaFold, and CASP13 winner RaptorX (group 498).

**Figure 3:**
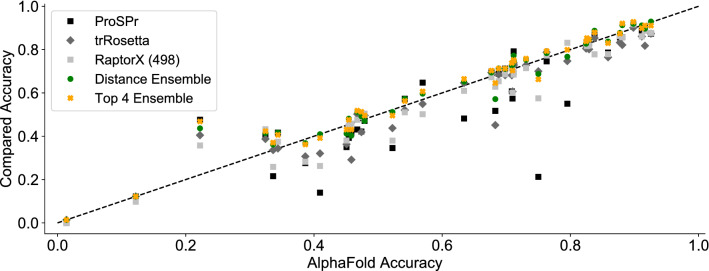
Scatterplot comparing accuracies of long-range contact predictions made by AlphaFold against a variety of other methods for 41 CASP13 targets, including the individual methods ProSPr, trRosetta, and Raptor X (group 498) as well as two ensembles. The Distance Ensemble combines predictions from ProSPr, trRosetta, and AlphaFold. The Top 4 Ensemble uses predictions from the top 4 individual methods—ranked by average accuracy—which are the three networks included in the Distance Ensemble, with the addition of RaptorX (498).

In a next step we combined the three post-CASP distance prediction networks into a “Distance Network (DN) Ensemble”, which results are also shown in Table 1. Figure 3 compares the accuracy of contact predictions made by the DN Ensemble with those made by AlphaFold for the test set of 41 CASP13 targets. While the ensemble is inferior in rare cases, in most instances, and on average, the accuracies of DN Ensemble predictions are superior to those made by AlphaFold alone—another evidence for the superiority of ensembling.

To take this idea a step further we investigated whether an ensemble of distance prediction methods and CASP13 entrants could result in further improvement of contact accuracy. Only group 498 (RaptorX) showed comparable accuracies in all three categories with the post-CASP13 distance-based methods, therefore we constructed an Overall Top 4 Ensemble consisting of AlphaFold, trRosetta, ProSPr, and RaptorX (498). The average accuracy scores are shown in Table 1 and compared with AlphaFold for each target in Fig. 3. We observe that this ensemble outperforms the previous best method in average mid, long, and overall accuracies on the test set.

### Value the differences–why ensembling helps

The question arises, why do ensembles perform better than individual method predictions? The definition of input vectors, network architectures, and training procedures has a significant impact on the ability of a neural network to generalize beyond the data set that it was trained on. CASP targets generally make up very interesting test sets, as they often contain novel protein folds. It is important to note that all ensembled methods had comparable accuracies on the test set, while the predictions themselves were dissimilar, as quantified by the Jaccard distances between 0.355 and 0.471 (see Table 2).

**Table 2:** Jaccard distances for pairs of deep learning distance prediction networks and the top performing CASP13 contact prediction group (RaptorX) for each contact category.

Method Pair		Short	Mid	Long	Average
ProSPr	trRosetta	0.361	0.401	0.463	0.408
ProSPr	AlphaFold	0.355	0.413	0.452	0.407
trRosetta	AlphaFold	0.385	0.408	0.463	0.419
RaptorX (498)	ProSPr	0.374	0.420	0.480	0.425
RaptorX (498)	trRosetta	0.382	0.415	0.471	0.422
RaptorX (498)	AlphaFold	0.390	0.405	0.444	0.413

A detailed comparison of network architectures is out of scope for this report, but the reported methods represent deep residual neural networks and mainly differ with respect to input vectors and auxiliary predictions. The importance of input vectors is currently a topic of active research, as networks appear to be very dependent on high quality multiple sequence alignments and associated features generated for a given protein sequence. Additionally, a common problem in supervised training is overfitting, and some of the false positive predictions made by the networks might be the result of overly confident assignment of probabilities.

It is important to point out that the greatest differences between the methods are among the long contact predictions, which correspond to the contact category that benefited most from ensembling. Therefore, dissimilar network models with similar prediction accuracies can be expected to benefit most from ensembling, as this likely mitigates the errors of individual networks, such as overfitting. Predictions, like those of AlphaFold, are in themselves already the result of ensembling multiple networks, but the differences were probably not sufficiently large to yield the same improvements observed for more diverse ensembles in this study.

Averaging over the ensemble components increases the contact probabilities associated with agreeing predictions and downweighs contacts that are only predicted by a single network. This effective reweighting of contact probabilities in an ensemble might be comparable to error reduction through repeated measurements. As long as the ensembled predictions are of comparable quality, the ensemble tends to outperform the individual contributions—or the whole is greater than its parts.

### Applying the ensemble–contact sensitivity to mutations

**Figure 4:**
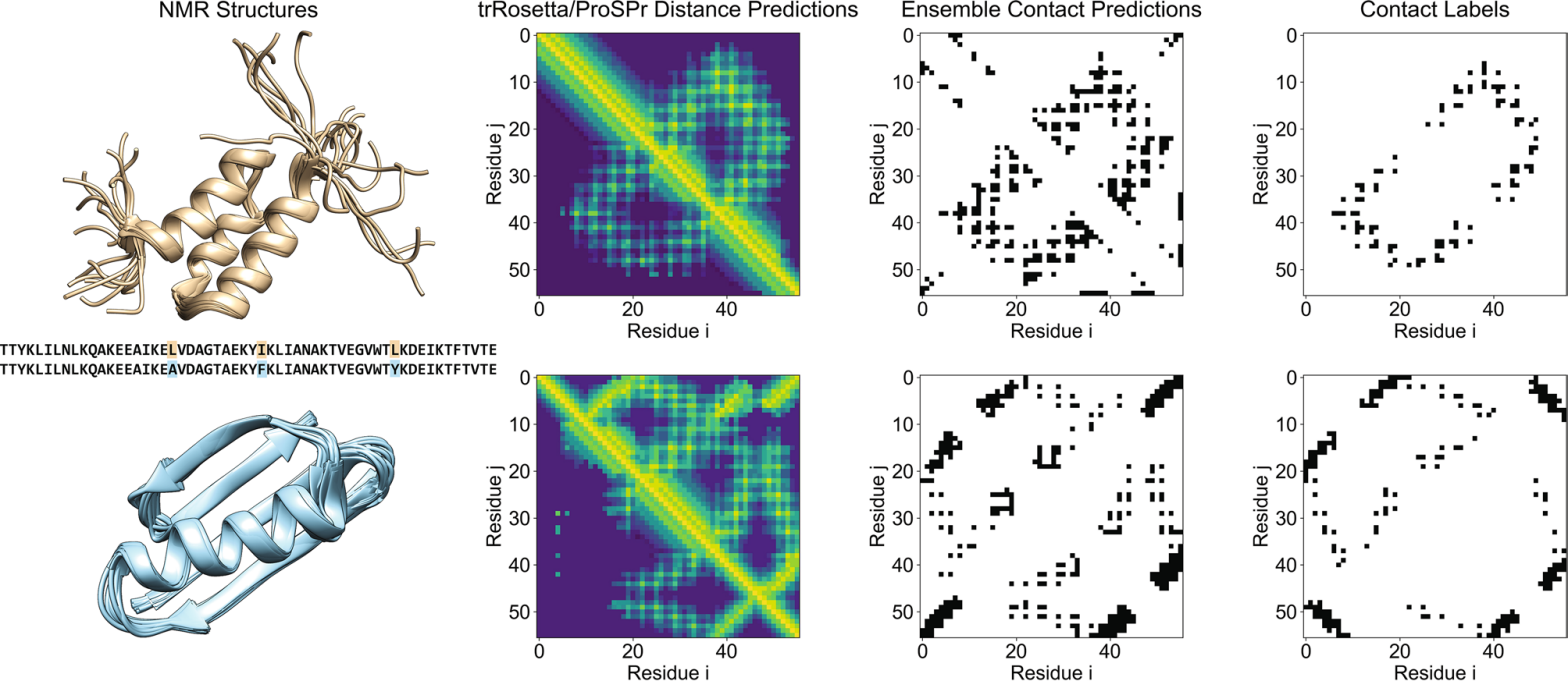
PDB 2KDL (first row) and 2KDM (second row) case example. The first column depicts the overlaid NMR structures reported in the PDB and the sequence, highlighting mutation points. The second column shows the distance predictions made by trRosetta in the bottom left half of the plot contrasted with ProSPr predictions in the top right diagonal. The third column shows the ensembled trRosetta and ProSPr contact predictions. The final column depicts the contact labels derived from the NMR structures.

In order to apply our findings to a relevant example, we ensembled the most accessible deep learning networks: trRosetta and ProSPr. The performance of this ensemble on the CASP13 dataset was better than each individual method and on average 2% inferior to the ensemble that also contained AlphaFold. As an example, we selected two famous NMR models (PDB: 2KDM and PDB Id: 2KDL) whose sequences only differ by three point mutations but fold very differently^22^ (see Fig. 4). For these sequences, trRosetta contact predictions yielded average accuracies of 85.38 and 55.52%. After ensembling the predictions with ProSPr (see Method “Applying the ensemble to a test case of two similar sequences” section), the accuracies improved over trRosetta by 1.75 and 8.77%, respectively. This shows that deep learning methods can predict different folds for very similar sequences. Further, ensembling of two contact predictors yielded superior results compared to trRosetta alone. The ability to predict large structural changes due to few mutations holds great promise for protein engineering, for example to identify conserved residues whose mutations would distort the fold of a catalytic site.

## Conclusion

We created multiple ensembles combining groups that participated in CASP13 contact prediction, as well as several recent deep networks to demonstrate the usefulness of ensembling for protein contact prediction. We found that similar to successes observed in the machine learning literature^9^, ensembling improves contact predictions if the ensembled methods are different and by themselves of high quality. We also showed that the success of this technique extends to an example of very similar sequences that adopt different folds, which holds promise for protein engineering. For this ensembling method to be meaningfully applied, the following two issues should first be resolved.

First, it is necessary to gauge new and existing contact predictors against a standard benchmark. In order to rank predictions, a test set is needed that was not used for training the respective methods. Here we used structures from CASP13 as a test set, as training of all methods which were ensembled predated the availability of the associated structures. It was a large effort to find, clean, and use the CASP13 dataset, and even two years after the fact, only 41 targets could be prepared. As time progresses and new methods emerge, we can assume that other networks will be trained on structures which are a subset of CASP13 data. However, we require a test set on which all methods can be benchmarked—without training bias—to rank and select a set of predictors for ensembling. We therefore encourage the community to provide a test dataset and training set similar, but in higher quantity and quality, to Badri et al*.*^23^ In the interim, we point the community to the training set used for ProSPr^17^ and the CASP13 label set (see Data Availability) used in this study as a starting point. Efforts similar to those of Shapovalov et al.^24^ that provide test sets for protein secondary structure prediction might also form a basis that can be expanded into larger databases.

Second, any good new network has the potential to strengthen an ensemble, but this can only be realized if the prediction method is made easily accessible to the user. We observed that ensembling benefits from different architectures and networks even if they are slightly inferior to existing solutions. Contrary to the publication bias, we urge the community to make useable contact or distance prediction networks available, even if they cannot quite see eye to eye with the current state-of-the-art solutions. Combining new networks with existing methods could lead to an ever-improving contact prediction tool, and unparalleled protein structures in the future.

The best ensemble found in this study outperformed protein contact predictions of the best stand-alone solution, AlphaFold, on the CASP13 test set by an average of 1.15%. Because models like AlphaFold are difficult to reuse, others will benefit most from these findings if more models are made publically available in an easy-to-use fashion, such as trRosetta or ProSPr. We hope to find many more usable networks in the near future, as their contributions as parts will create a whole of greater value for the community.

## Data Availability

CASP13 target structures and labels, ProSPr predictions, and trRosetta predictions are made available in the data archive SimTK (https://simtk.org/projects/ensemble). Also included are the labels, alignment files, and distance predictions made by ProSPr and trRosetta for the 2KDL/2KDM test example.
